# Developing a Wi-Fi Sensing-Based Deep Learning Solution for Recognizing Daily Activities in Older Adults

**DOI:** 10.1093/geroni/igaf122.1770

**Published:** 2025-12-31

**Authors:** Jane Chung, M D Touhiduzzaman, Ingrid Pretzer-Aboff, John Karlsen, Megan Vain, Eyuphan Bulut

**Affiliations:** Emory University, Atlanta, Georgia, United States; Virginia Commonwealth University, Richmond, Virginia, United States; Virginia Commonwealth University, Richmond, Virginia, United States; Virginia Commonwealth University, Richmond, Virginia, United States; Virginia Commonwealth University, Richmond, Virginia, United States; Virginia Commonwealth University, Richmond, Virginia, United States

## Abstract

Assessing older adults’ daily activities and detecting early changes is important for identifying those at risk of functional decline and enabling timely interventions. Given the limitations of existing sensing technologies, there is a need for discreet, affordable system that quantifies daily activity levels and patterns with minimal user engagement. We aimed to develop a Channel State Information (CSI)-based device-free Wi-Fi sensing system using deep neural network (DNN) classification models to localize and recognize different in-home activities in older adults. We deployed three transmitter-receiver pairs in participants’ residences and collected Wi-Fi CSI data over 5-7 days. Ground truth annotations for activities such as entering or exiting the residence, entering or exiting a bedroom, working in the kitchen, and opening or closing the refrigerator were derived from video recordings. We trained DNN models with a one-dimensional convolutional layer for each transmitter-receiver pair. To improve accuracy, we combined predictions from different pairs using class-weighted linear regression. Finally, we applied majority voting across consecutive predictions to enhance reliability. The prediction accuracy provided by our models using the six activities ranges between 75-90% for different participants (average accuracy = 83%). Participants generally had no to minimal concerns about privacy and obtrusiveness but differed in their views on the system’s usefulness, due to the lack of clear information on how the sensing data could benefit their health and wellbeing. This sensing system’s ability to distinguish human activities has the potential to enable early detection of functional decline, a potential indicator of cognitive impairment.

